# RELATIONSHIP BETWEEN THE PRESENCE OF *HELICOBACTER PYLORI* WITH INFLAMMATORY ENDOSCOPIC CHANGES IN GASTRODUODENAL MUCOSA

**DOI:** 10.1590/0102-6720201600030004

**Published:** 2016

**Authors:** Irma Cláudia Saboya RIBEIRO, Luiz Fernandao KUBRUSLY, Paulo Afonso Nunes NASSIF, Patrícia Fernanda Saboya RIBEIRO, Rodrigo de Oliveira VERAS, Aline NEPPEL

**Affiliations:** 1 Postgraduate Program in Principles of Surgery, Evangelic Faculty of Paraná/University Evangelic Hospital of Curitiba/Medical Research Institute, Curitiba, PR, Brazil; 2 Gastrointestinal Endoscopy Service, 9 of July Hospital, São Paulo, SP, Brazil

**Keywords:** Endoscopy, Esophagitis, Gastrites, Duodenitis, Helicobacter pylori, Inflammation

## Abstract

**Background:**

The influence of Helicobacter pylori (HP) in inflammatory disorders of the digestive mucosa has been the subject of several studies since socioeconomic, personal and environmental factors were implicated in the bacteria transmission.

**Aim:**

To correlate the inflammatory endoscopic findings with HP infection and the onset of mucosal diseases mucous of the upper digestive tract.

**Method:**

Comparative observational study, in which were collected data from 2247 patients who underwent upper endoscopy and biopsies for HP with urease test. The patients were divided into two groups: HP+ and HP- (control) in which endoscopic findings were observed for the following changes: esophagitis, esophageal ulcer, gastritis, erosive gastritis, gastric ulcer, bulboduodenitis, bulbar ulcer and without disease.

**Results:**

As for esophagitis, there was little disparity in the distribution favorable to HP+ group (HP+ =67.11% and HP- =69.89%) and esophageal ulcer (HP+ =0% and HP- =0, 21%). Gastritis was favorable to HP- group (HP+ =78.34% and HP- =73.63%), as well as erosive gastritis (HP+ = 67,11% and HP- = 64,55%), in bulboduodenitis (HP+ =1,87% and HP- 1,23%), in gastric ulcer (HP+ =2,14% and HP- =2,03%) and in the absence of alterations in the HP+ group (4.81%) with the HP- control group (6,30%), in which there was little disproportion in favor of HP- group, but without statistical significance. As for the bulbar ulcer (HP +=10.16% and HP- =4.48%), there was statistically significant (p=0.00001).

**Conclusion:**

There is no difference between HP+ and HP- groups in inflammatory changes in endoscopic gastroduodenal mucosa, except for the relationship between HP and bulbar ulcer.

## RESUMO

**Racional:**

A influência do Helicobacter pylori (HP) nas alterações inflamatórias das mucosas digestivas tem sido objeto de vários estudos uma vez que fatores socioeconômicos, pessoais e ambientais são implicados na transmissão da bactéria, facilitando-a.

**Objetivo:**

Relacionar os achados inflamatórios endoscópicos com a infecção pelo HP e o aparecimento de doenças mucosas do trato digestivo alto.

**Método:**

Estudo observacional comparativo, o qual foram coletados dados de 2247 pacientes submetidos à endoscopia digestiva alta e biópsias para HP com teste de urease. Os pacientes foram divididos em dois grupos: HP+ e o controle HP-dentro dos quais foram observados os achados endoscópicos referentes às seguintes alterações: esofagite, úlcera esofágica, gastrite, gastrite erosiva, úlcera gástrica, bulboduodenite, úlcera bulbar e sem doença.

**Resultados:**

Quanto à esofagite, observou-se pequena desproporção na distribuição, favorável ao grupo HP+ (HP+ =67,11% e HP- =69,89%) bem como na úlcera esofágica (HP+ =0% e HP- =0,21%). Na gastrite foi favorável ao grupo HP- (HP+ =78.34% e HP- =73.63%), assim como na gastrite erosiva (HP+ = 67,11% e HP- = 64,55%), na bulboduodenite (HP+ = 1,87% e HP- 1,23%), na úlcera gástrica (HP+ =2,14% e HP- =2,03%) e na ausência de alterações no grupo HP+ (4.81%) com o grupo controle HP- (6,30%), nos quais há pequena desproporção favorável ao grupo HP-, porém, sem significância estatística. Já quanto à úlcera bulbar (HP+ =10,16% e HP- =4,48%), houve significância estatística (p=0,00001).

**Conclusão:**

Não há diferenciação entre os grupos HP+ e HP- nas alterações endoscópicas inflamatórias na mucosa gastroduodenal, exceto para a relação entre HP e úlcera bulbar.

## INTRODUCTION

The discovery of *Helicobacter pylori* (HP) and the analysis of its correlation with inflammatory changes of the digestive mucosa has been the subject of numerous studies. Many points correlate this bacterium and wide range of gastroduodenal diseases by modifying the understanding of the pathophysiology and creating open questions whether its treatment can prevent complications.

It is known that the acquisition of this infection has interpersonal transmission, is related to socioeconomic and environmental factors, being more prevalent in developing countries^4^ ^,^ ^18^ ^,^ ^26^.

HP is gram-negative spiral bacillus, flagellate, usually not invasive, remaining on the surface of the gastric mucosa. Small proportion of the bacterial cells adheres to mucosal epithelium. Its spiral shape and flagella make it mobile on the mucosal environment and its effective urease protects against acid, catalyzing urea hydrolysis to produce ammonia buffer^7^.

HP infection is related to the development of lesions and gastric lymphomas; however, it is not known for sure if there is a correlation with gastroesophageal reflux and reflux esophagitis^3^ ^,^ ^20^. The literature already described its association with duodenitis^6^ ^-^ ^8^ and functional dyspepsia^5^ ^,^ ^8^ ^-^ ^13^.

Establish properly the relationship with the various gastroduodenal diseases is challenging, because the presence of infection has an influence in the same patient positively in some aspects and negative in others.

The aim of this study was to correlate the inflammatory endoscopic findings in positive and negative HP groups in order to examine whether there is a relationship with the onset of major mucosal diseases of the upper digestive tract.

## METHODS

It is a comparative observational study. Data were collected from 2247 patients undergoing endoscopy and biopsies for HP with urease test attended by the Endoscopy Service, Hospital 9 of July, São Paulo, SP, Brazil. 

Inclusion criteria were patients over 18 years of both genders who underwent endoscopy with rapid urease test, regardless of the examination reason request, ongoing disease or drug use. Exclusion criteria were: active bleeding during the examination; use of anticoagulants; without HP result by urease; HIV positive; with previous operations; tumors in the esophagus, stomach and duodenum; and when full and detailed examination was not possible (resistant to sedation, hypoxia or arrhythmia during the examination).

Based on 2247 patients was formed two groups: HP+ 16.6% and HP- with 83.4%. Within them, were searched endoscopic findings for the following alterations: esophagitis according to the Los Angeles classification, esophageal ulcer, non-erosive gastritis according to the Sydney classification, erosive gastritis, gastric ulcer, bulboduodenitis, bulbar ulcer and without disease. To identify ulcers, was used the definition of Paris classification.

For the urease and/or histological exams, biopsies were performed on body and antrum with biopsy forceps, and the material was placed in prefabricated reagent for the urease test.

The patients were previously submitted to the usual preparation for endoscopy - fasting for 8 h for solids and liquids. Immediately before the test were asked to ingest 10 ml of water with 40 drops of simethicone and, also, submitted to 5-10 puffs of lidocaine spray in the oropharynx.

Statistical analysis

For the analysis of the data was used the statistical software SPSS 23.0. Patients whose urease test was negative (HP-) served as control group for those who test was positive (HP+) in order that it could compare the HP presence influenced positively or negatively the studied conditions. To determine whether the samples had statistically significant results, was used the Pearson chi-square with an accuracy of 5% or p<0.5, in order to avoid results that could have been generated at random.

## RESULTS

### Comparative analysis of mucosal changes

#### Esophagitis in HP+ and HP- groups

Comparing the incidence of esophagitis between HP+ group (67.11%) with the HP- control group (69.89%) there was little disparity in the distribution favorable to HP+ group that had slightly lower percentile of this disease. However, the data analysis about this disproportion was not statistically significant (p=0.287464, >.05, Table 1A)

**TABLE 1:** Incidence of esophagitis (A) and esophageal ulcer (B) in the HP+ and HP- groups

A		esophagitis yes	%	esophagitis no	%	total	%
	Hpylori positive	251	67,11	123	32,89	374	100,00
	Hpilory negative	1309	69,89	564	30,11	1873	100,00
	total	1560	69,43	687	30,57	2247	100,00
B		esopha ul yes	%	esopha ul no	%	total	%
	Hpylori positive	0	0,00	374	100	374	100,00
	Hpilory negative	4	0,21	1869	99,79	1873	100,00
	total	4	0,18	2243	99,82	2247	100,00

#### Esophageal ulcer in HP+ and HP- groups

Comparing the incidence of esophageal ulcer in HP+ group (0%) with the HP- (0.21%) was observed low incidence in both groups, with small disproportion in favor distribution to HP+ group that showed no cases of this disease. However, analysis of the disproportion was not statistically significant (p=0.371051, >.05, Table 1B).

#### Gastritis in HP+ and HP- groups

Comparing the incidence of gastritis in HP+ group (78.34%) with the HP- control group (73.63%) there was a certain disproportion in the distribution, in favor of HP-group that had lower percentile. However, the data analysis of this disproportion was not statistically significant (p=0.56, >.05, Table 2).

**TABLE 2:** Gastritis incidence on HP+ and HP- groups

	gastritis yes	%	gastritis no	%	total	%
Hpylori positive	293	78,34	81	21,66	374	100,00
Hpilory negative	1379	73,63	494	26,37	1873	100,00
total	1672	74,41	575	25,59	2247	100,00

#### Erosive gastritis in HP+ and HP- groups

Comparing the incidence of erosive gastritis in HP+ group (67.11%) and HP- (64.55%) there was little distribution disproportion in favor to the HP- group showing slightly lower percentile. However, the data analysis of this disproportion was not statistically significant (p=0.343, >.05, Table 3).

**TABLE 3:** Effect of erosive gastritis in HP+ and HP- groups

	eros gast yes	%	eros gast no	%	total	%
Hpylori positive	251	67,11	123	32,89	374	100,00
Hpilory negative	1209	64,55	664	35,45	1873	100,00
total	1460	64,98	787	35,02	2247	100,00

#### Bulboduodenitis in HP+ and HP- groups

Comparing the incidence of bulboduodenitis in HP+ and HP- groups (1.23%) was observed small disproportion in favor distribution to HP that showed slightly lower percentile group. However, the data analysis of this disproportion was not statistically significant (p=0.322, >.05, Table 4).

**TABLE 4:** Bulboduodenitis incidence on HP+ and HP- groups

	bulboduo yes	%	bulboduo no	%	total	%
Hpylori positive	7	1,87	367	98,13	374	100,00
Hpilory negative	23	1,23	1850	98,77	1873	100,00
total	30	1,34	2217	98,66	2247	100,00

#### Gastric ulcer in HP+ and HP- groups

Comparing the incidence of gastric ulcer in HP+ group (2.14%) and HP- (2.03%) there was little disparity in the distribution, in favor to HP- group showing slightly lower percentile. However, the data analysis of this disproportion was not statistically significant (p=0.523, >.05, Table 5).

**TABLE 5:** Incidence of gastric ulcer in HP+ and HP- groups

	gastr ul yes	%	gastr ul no	%	total	%
Hpylori positive	8	2,14	366	97,86	374	100,00
Hpilory negative	38	2,03	1835	97,97	1873	100,00
total	46	2,05	2201	97,95	2247	100,00

#### Bulbar ulcer in HP+ and HP- groups

Comparing the incidence of bulbar ulcer in HP+ group (10.16%) with the HP-control group (4.48%) was observed inequalities in the distribution, in favor of HP-group that had lower percentile. Furthermore, the analysis of data from this disproportion was highly statistically significant (p=0.00001, <.05, Table 6A).

**TABLE 6:** Incidence of bulbar ulcer (A) and absence of lesions (B) on HP+ and HP- groups

A		bulbar ul yes	%	bulbar ul no	%	total	%
	Hpylori positive	38	10,16	336	89,84	374	100,00
	Hpilory negative	84	4,48	1789	95,52	1873	100,00
	total	122	5,46	2125	94,57	2247	100,00
B		no alter yes	%	no alter no	%	total	%
	Hpylori positive	18	4,81	356	95,19	374	100,00
	Hpilory negative	118	6,30	1755	93,70	1873	100,00
	total	136	6,05	2111	93,95	2247	100,00

#### Absence of alterations in the HP+ and HP- groups

Comparing the incidence of the absence of changes in HP+ group (4.81%) with the HP- control group (6.30%) there was a certain distribution disproportion in favor to the HP- group that presented percentile a little larger than absence of disease. However, the data analysis of this disproportion was not statistically significant (p=0.2708, >.05, Table 6B).

## DISCUSSION

Colonization by HP mucosa predisposes to peptic ulcer disease, gastric atrophy and subsequent gastric cancer due to various changes in gastric acid secretion.

In this study the most common diseases for patients with PH+ were gastritis (78.34%), esophagitis (67.11%) and erosive gastritis (67.11%). Other diseases have very low prevalence and were bulbar ulcer (10.16%), gastric ulcer (2.14%), bulboduodenitis (1.87%) and esophageal ulcer (0.00%), with similar results already presented^3^ ^,^ ^5^ ^,^ ^17^ ^,^ ^23^.

Although the bacteria was initially implicated in the pathogenesis of gastroesophageal reflux and esophagitis, several studies have shown that its prevalence is smaller than the rest of population^19^ ^,^ ^22^ ^,^ ^25^ ^,^ ^28^. It is explained by gastric atrophy and subsequent reduction of gastric acid secretion, which would lead to lower esophageal injury. Nevertheless, due to its involvement in gastric carcinogenesis is recommended its erradication^15^ ^,^ ^19^.

It was not analyzed here the carcinogenic effects, direct or indirect, nor gastric atrophy or gastroesophageal reflux associated with infection by HP. It was possible to analyze data related to ulcers in the digestive tract, where it was confirmed a direct relationship between the presence of ulcers and HP+; but only to bulbar ulcers this ratio proved to be significant. With no correlation with the literature, was observed the inverse relationship between HP and esophageal ulcer, but with very small and statistically insignificant disproportion.

The data also demonstrated an inverse relationship between the presence of HP and esophagitis, with smaller percentage for HP+ group compared to HP-. The numbers of this relationship did not reach statistical significance, but confirmed the cited literature.

The HP infection is considered the main cause of chronic gastritis^1^ ^,^ ^9^ and studies suggest that this agent plays an important role in the genesis of peptic ulcer^19^. After finding that its eradication entails the healing of the ulcer, it was decided that patients with HP should receive specific treatment. The eradication of bacteria restores the gastric mucosa and end the carcinogenic development of acute gastritis (chronic gastritis - atrophy - metaplasia - dysplasia - cancer). Muller et. al.^12^ found that patients with HP+ showed odds ratio 10 times higher (95% CI=6.50% -17%) to present some degrees of gastric mucosal injury than those with absence. It is estimated that only portion of infected individuals develop HP gastroduodenal diseases due to different pathogenic strains. Studies have shown that infection with HP- Cag A+ is associated with alterations in cell cycle and apoptosis in gastric epithelial and more pronounced levels of inflamation^11^ ^,^ ^14^. The HP Cag A+ is also associated with higher intensity of atrophy and intestinal metaplasia^10^ ^,^ ^16^ ^,^ ^21^ ^,^ ^24^.

Studies have also shown a strong relationship between duodenal ulcer and HP^26^ ^,^ ^27^. They found duodenal ulcer as the most common injury in their papers. Caetano et. al.^2^ studied 150 patients prospectively evaluating, from the total of 1,043 cases, chronic gastritis that was found in 109 patients (72.6%), gastric ulcer in six (4%), nine chronic duodenitis (6%) and 26 duodenal ulcers (17.4%). Then, they grouped patients in which 103 (68.67%) had positive urease test, 104 (69.33%) histologically positive and 98 (65.33%) seropositivity for HP. Marques^12^, analyzing 1478 presented the most frequent endoscopic findings: gastritis (n=810, 54.8%); gastric and duodenal peptic ulcers (n=494, 33.4%); duodenitis (n=287, 19.4%) and esophagitis (n=217, 14.7%); prevalence of HP infection in 53% (n=786) increasing the risk of gastric and duodenal ulcers in 1.9 and 1.6 times.

The data presented here had much lower percentage than reported in the literature (16.6%). However, they denote certain propensity, although small and insignificant, of the HP+ individuals to present changes in mucosal group, and on a global analysis, only 6.30% of HP+ group did not show any kind of change against 4.81% the HP- (control) group. It must be emphasized, again, that for specific relationship between HP and ulcer bulbar the numbers were significant.

## CONCLUSION

There is no differentiation between HP- and HP+ groups in endoscopic inflammatory changes in the gastroduodenal mucosa, except for the relationship between HP and bulbar ulcer.
